# Redox responses are preserved across muscle fibres with differential susceptibility to aging

**DOI:** 10.1016/j.jprot.2018.02.015

**Published:** 2018-04-15

**Authors:** Neil T. Smith, Ana Soriano-Arroquia, Katarzyna Goljanek-Whysall, Malcolm J. Jackson, Brian McDonagh

**Affiliations:** a MRC-Arthritis Research UK Centre for Integrated Research into Musculoskeletal Ageing, Department of Musculoskeletal Biology, Institute of Ageing and Chronic Disease, University of Liverpool, Liverpool L7 8TX, UK; b Discipline of Physiology, School of Medicine, National University of Ireland Galway, Ireland

**Keywords:** Skeletal muscle, Aging, Redox proteomics, Cysteine oxidation, Sarcopenia, skNAC

## Abstract

Age-related loss of muscle mass and function is associated with increased frailty and loss of independence. The mechanisms underlying the susceptibility of different muscle types to age-related atrophy are not fully understood. Reactive oxygen species (ROS) are recognised as important signalling molecules in healthy muscle and redox sensitive proteins can respond to intracellular changes in ROS concentrations modifying reactive thiol groups on Cysteine (Cys) residues. Conserved Cys residues tend to occur in functionally important locations and can have a direct impact on protein function through modifications at the active site or determining protein conformation. The aim of this work was to determine age-related changes in the redox proteome of two metabolically distinct murine skeletal muscles, the quadriceps a predominantly glycolytic muscle and the soleus which contains a higher proportion of mitochondria. To examine the effects of aging on the global proteome and the oxidation state of individual redox sensitive Cys residues, we employed a label free proteomics approach including a differential labelling of reduced and reversibly oxidised Cys residues. Our results indicate the proteomic response to aging is dependent on muscle type but redox changes that occur primarily in metabolic and cytoskeletal proteins are generally preserved between metabolically distinct tissues.

**Biological significance:**

Skeletal muscle containing fast twitch glycolytic fibres are more susceptible to age related atrophy compared to muscles with higher proportions of oxidative slow twitch fibres. Contracting skeletal muscle generates reactive oxygen species that are required for correct signalling and adaptation to exercise and it is also known that the intracellular redox environment changes with age. To identify potential mechanisms for the distinct response to age, this article combines a global proteomic approach and a differential labelling of reduced and reversibly oxidised Cysteine residues in two metabolically distinct skeletal muscles, quadriceps and soleus, from adult and old mice. Our results indicate that the global proteomic changes with age in skeletal muscles are dependent on fibre type. However, redox specific changes are preserved across muscle types and accompanied with a reduction in the number of redox sensitive Cysteine residues.

## Highlights

• Skeletal muscle fast twitch fibres are particularly susceptible to age-related atrophy.
• Proteomic changes with age in skeletal muscles are dependent on fibre type.
• Redox specific changes are preserved across fibres with different susceptibility to age-related atrophy.
• Decreased number of redox sensitive Cysteine residues detected with age in all muscle fibres.

## Graphical abstract

**Figure:**
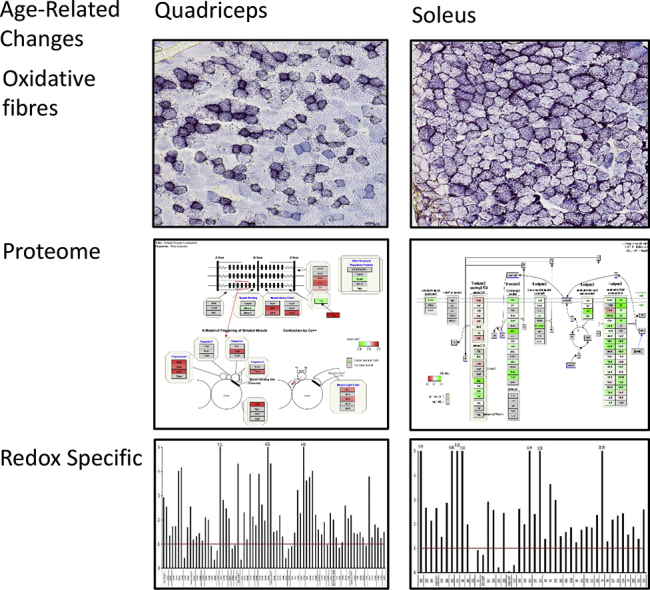

## 1 Introduction

Skeletal muscle is the largest organ in the body accounting for up to 50% of total body mass. The age-related loss of skeletal muscle mass and function can have a major impact on quality of life and result in loss of independence and an increase in frailty. Skeletal muscle is composed of a mixture of fibre types that can be broadly categorised into fast and slow twitch fibres depending on their myosin heavy chain content. Quadriceps muscle is generally referred to as a fast twitch muscle that primarily produces ATP via glycolysis [1]. The soleus, on the other hand, predominantly uses mitochondrial dependent oxidative phosphorylation via the electron transport chain for ATP generation. In mice the quadriceps is considered a glycolytic muscle and in common with similar muscles such as the tibialis anterior, has a high proportion of type IIB fibres (~60%) while the soleus has a higher proportion of oxidative Type I (~37%) and IIA (~38%) fibres [2]. Human and rodent studies have revealed that fast twitch fibres such as the quadriceps are more susceptible to age-related atrophy than their slow-twitch counterparts such as soleus [[3], [4], [5]]. Nevertheless age-related loss of mitochondrial content and altered mitochondrial morphology has been reported in all skeletal muscle [[6], [7], [8], [9]].

Reactive oxygen species (ROS) are involved in the degeneration of muscle with age but it is unclear how this affects different fibre types [10]. In skeletal muscle there are a number of potential sites for ROS generation including electron leakage from the electron transport chain and endogenous generators of ROS species such as NADPH oxidase (NOX) enzymes that are activated by muscle contractions [[11], [12], [13]]. Although ROS were traditionally thought to be responsible for oxidative damage more recent evidence would suggest that they also play a crucial signalling role within the cell that is dependent on the location, species and concentration of ROS generated [14]. A number of recent studies have identified a beneficial role of exercise induced ROS in redox signalling that is required for the adaptation and repair of injured skeletal muscle [15,16]. ROS are primarily thought to mediate their signalling activities through the selective oxidation/reduction of specific Cysteine (Cys) residues on target proteins. Cys residues have a functionally important thiol group which generally has a low mean p*K*_a_ and exist as nucleophiles at physiological pH, although it is dependent on the local environment which can impact Cys reactivity [17]. Reversible oxidation of Cys residues into sulfenic acids (-SOH) and subsequent formation of disulfide bonds (-S-S-) between thiol groups can potentially alter the functional role of sensitive proteins by changing their conformation and affecting their activity or metal ion binding [18,19]. Further oxidation of thiols via sustained exposure to ROS can lead to the formation of sulfinic (-SO_2_H) and sulfonic acids (-SO_3_H). Reversible modifications of Cys residues enable these residues to participate in local redox signalling events in response to ROS, however a sustained increase in ROS over time can potentially lead to irreversible modifications.

Skeletal muscle from old mice have a blunted response to the adaptations induced by exercise in part due to an altered intracellular redox environment [20]. A chronically elevated intracellular ROS environment would modify redox sensitive proteins in skeletal muscle affecting redox signalling and the adaptive response to exercise. In this study we have utilised a global label free and redox proteomic approach [21] to identify the age related redox proteomic changes in two skeletal muscle types that differ in their primary sources of energy for contraction, quadriceps and soleus from adult and old mice. The redox state of individual Cys residues was calculated using the parent ion intensities of Cys containing peptides labelled with either a light (reduced Cys residues and labelled with *d*0 N-Ethylmaleimide (NEM)) or heavy (reversibly oxidised labelled Cys residues and labelled with *d*5 NEM) alkylating agent. Our results demonstrate that the majority of proteins identified between two metabolically distinct muscles was consistent but the effects of age on the protein composition of the two muscle groups was very different. In glycolytic quadriceps tissue there was an increase in cytoskeletal proteins with age and in oxidative soleus a decrease in mitochondrial related proteins. In contrast redox analysis of Cys residues indicated that in both tissues the number of Cys residues labelled as reduced and reversibly oxidised decreased with age, but that the core redox sensitive proteins involved in metabolic processes are preserved between muscle types.

## 2 Materials and methods

All reagents and chemicals unless otherwise stated were obtained from Sigma Aldrich (Dorset, UK) and were of analytical grade or above.

### 2.1 Animals

Adult (12 months) and old (25 months) C57BL/6 male mice were purchased from Charles River and housed in the Specific Pathogen-Free (SPF) Facility at the University of Liverpool for at least 2 weeks before use. Experiments were performed in accordance with U.K. Home Office Guidelines under the U.K. Animals (Scientific Procedures) Act 1986 and received ethical approval from the University of Liverpool Animal Welfare and Ethical Review Board. Body and muscle weights were compared using a two tailed Student's *t*-test. Animals were sacrificed by cervical dislocation, white glycolytic quadriceps tissue and whole soleus muscle were dissected and placed immediately in thiol blocking buffer containing 25 mM *d*0 N-Ethylmalemide (NEM) and 50 mM ammonium bicarbonate, pH 8, for redox proteomic analysis. The contralateral muscles were dissected, orientated transversely, mounted on a cork block and frozen in optimal cutting temperature compound (OCT) for subsequent histological examination.

### 2.2 Succinate dehydrogenase staining

Dissected skeletal muscles (quadriceps or soleus at either 12 or 25 months of age), that were transversely orientated in OCT were moved from −80 °C storage to the Cryostat (Leica, Milton Keynes, UK) to acclimatise to −20 °C. Cryo sections were cut at a thickness of 10 μm and stained for succinate dehydrogenase (SDH) activity as described [22]. Histological analysis was performed on a Zeiss Axiovert 200M microscope connected to a PC running Windows XP and Axiovision 4.8.2 (SP3 08-2013). Images were taken using Axiocam ICc5 under bright field conditions at 10×/0.30 magnification for an exposure time of 2 ms giving a 2452 × 2056 pixel image with scale factors of x 0.55 μm/pixel, y 0.55 μm/pixel and z 1.00 pixel/pixel. The % of stained fibres was calculated using a modified 3-point scale: 0 - unstained fibres; 1 – lightly stained fibres, 2 – dark stained fibres, for the semi quantification of SDH staining intensity as described previously [23]. The data shows % unstained/stained fibres of total fibres and analysed by two-way ANOVA followed by Bonferroni multiple comparison test (95% Confidence Interval), a *P*-value <0.05 was considered as statistically significant * *p* < 0.05; ** *p* < 0.01; *** *p* < 0.01. Statistical analysis was performed using GraphPad Prism version 5.01 for Windows (GraphPad Software, La Jolla California USA). All visible fibres in the section of the muscle were calculated using *N* = 3 biological replicates. Total SDH staining intensity was measured by mean pixel density using ImageJ software (National Institute of Health (Bethesda, USA)) from grayscale images manually traced at muscle boundaries as described previously [24].

### 2.3 Redox proteomics sample preparation

Sample preparation was performed as previously described [21], briefly each muscle (*N* = 5 for soleus tissue from adult and old mice and quadriceps tissue from old mice, *N* = 6 for quadriceps tissue from adult mice) was dissected and immediately placed in *d*0 NEM blocking buffer to prevent Cys oxidation. Tissues were homogenised in ice cold buffer (25 mM Ammonium bicarbonate containing 25 mM NEM and 0.1% Rapigest (Waters, Manchester, UK) pH 8) using a hand homogeniser. Samples were passed through a Zeba desalting column 7K MWCO (Thermo Scientific, Hempstead, UK) to remove excess *d*0 NEM and protein concentrations were calculated using the Bradford (BioRad, Hertfordshire, UK) method using BSA as a standard. 100 μg of protein was aliquoted and reversibly oxidised Cys residues were reduced using tris (2-carboxyethyl) phosphine (TCEP) at a final concentration of 10 mM, newly reduced Cys residues were subsequently labelled with *d*5 NEM final concentration 20 mM. Protein extracts were digested overnight at 37 °C with trypsin, Rapigest was precipitated by addition of trifluoroacetic acid (TFA) and subsequent centrifugation before analysis by MS.

### 2.4 Lc-MS/MS and label free quantification

Proteomics was performed using an Ultimate 3000 RSLC Nano system (Thermo Scientific) coupled to a Q-Exactive mass spectrometer (Thermo Scientific) as previously described. [21]. Detection of the peptides was performed by data dependent acquisition (DDA) which takes a select number of peptide peaks from the initial scan according to a rule set and the corresponding ions are then verified against this initial set via tandem mass spectrometry (MS/MS).

Raw spectra were converted to Mascot Generated Files (.mgf) using the Proteome Discoverer software (Thermo Scientific). The .mgf files were searched against the Uniprot mouse sequence database (*Mus musculus* database from 12th May 2012; 16,376 sequences) via an in-house Mascot database server (Matrix Science, London, UK). The search parameters were: peptide mass tolerances, 10 ppm; fragment mass tolerance, 0.01 Da, 1+, 2+ and 3+ ions; missed cleavages, 1; instrument type, ESI-TRAP. Variable modifications were included as: *d*0-NEM, *d*5-NEM, mono-, di-, and tri-oxidation of Cys residues and oxidation of methionine.

Label-free relative quantification software PEAKS7 (Bioinformatics Solutions Inc., Waterloo, Canada) was used to analyse RAW data files against the same mouse protein database for identifications with Mascot [25]. Normalisation was carried out using the total ion current and proteins were considered significantly changed between adult and old mouse protein samples using the following criteria: at least 3 unique peptide, −10 log P score of ≥20 (equivalent to a *p* value of 0.01), a fold change ≥1.5 and using a quality value of 0.8. PEAKS7 software includes a post-translational modification (PTM) algorithm applying the de novo sequencing module to search for a limited number of PTMs. All identified PTMs using this method adhere to the above search criteria and FDR validation.

Data visualization of significant changes from the label free proteomic data (max fold change ≥ 1.5 and –10logP ≥ 20) was performed using Perseus software and z-scoring of quantitative data for Heatmaps [26]. Label free data of all identified proteins was visualised using Volcano plots using Log_2_ (Fold Change) plotted against significance in R studio. Pathway analysis of label free quantitative proteomic data was performed using PathVisio [27] together with WikiPathways [28] to visualize and highlight altered pathways from identified proteins.

### 2.5 Targeted analysis of redox sensitive Cys residues

In these experimental conditions we considered Cys containing peptides that were identified as labelled with both *d*5-NEM and *d*0-NEM as redox sensitive Cys residues, reduced Cys were only labelled with *d*0-NEM and reversibly oxidised Cys were labelled with only *d*5-NEM. Redox Cys peptides were identified by detecting identical amino acid sequences containing *d*0 and *d*5 NEM independently and having a Mascot peptide score of >20. Peptides detected from Proteome Discoverer analysis of RAW files were selected for targeted analysis with the open software Skyline [29]. Targeted analysis applying *m*/*z*, retention times and fragmentation spectra for peptide selection allowed the calculation of the reduced/oxidised ratio (or *d*0/*d*5 NEM ratio) of the individual Cys residues using their parent ion intensities with Skyline [29]. For redox peptides the individual reduced/oxidised ratio for each redox Cys peptide was used to calculate an average ratio of specific Cys oxidised/reduced peptides. For identification of peptides, the result filtration parameters employed a false detection rate (FDR) of 1%, a mass error tolerance of 10.0 ppm and a retention time shift tolerance of 0.5 min. Search parameters for the MASCOT database defined peptide significance as equal to or greater than a MASCOT Score of 20.

The surrounding amino acid sequences (−6 to +6) of identified redox sensitive Cys residues identified were blasted against the Mus database to find potential common motifs in the surrounding amino acid residues using Motif-X [30]. In order to determine if particular molecular pathways were over-represented or potential protein-protein interactions between proteins identified as containing redox sensitive Cys residues String-DB was used [31].

### 2.6 Immunoblotting

Protein samples (20 μg) from three biological replicates were loaded on 10% or 12% gels for SDS PAGE and subsequently transferred to nitrocellulose membranes at 100 mA per gel over 1 h. Ponceau S staining of the membrane was used to insure equal protein loading. Membranes were blocked for 1 h with 3% non-fat dry milk in Tris buffered saline-tween (TBS-T) 1 h at room temperature. Membranes were incubated overnight at 4 °C with primary antibodies (HSC70 Stressgen SMC-104A, HSP70 Enzo ADI-SPA-810, PDI Abcam ab137110, SOD1 Enzo ADI-SOD-100, PRDX2 Abcam ab59539 and PRDX5 Abcam ab16944). Following washing with TBS-T membranes were incubated for 1 h with Horseradish peroxidase-conjugated secondary antibodies (anti-mouse and anti-rabbit IgG (Cell Signalling Technologies, Hitchin, UK). Peroxidase activity was detected using the enhanced chemiluminescence (ECL) kit (Amersham International, Cardiff, UK) and band intensities were analysed using ImageLab (v5; BioRad, US). Band intensities were normalised using corresponding Ponceau S stain for differences in protein loading and analysed using a two tailed Student's *t*-test.

## 3 Results

### 3.1 Age-related decrease in quadriceps tissue mass and alterations in SDH activity in both quadriceps and soleus

**Fig. 1:**
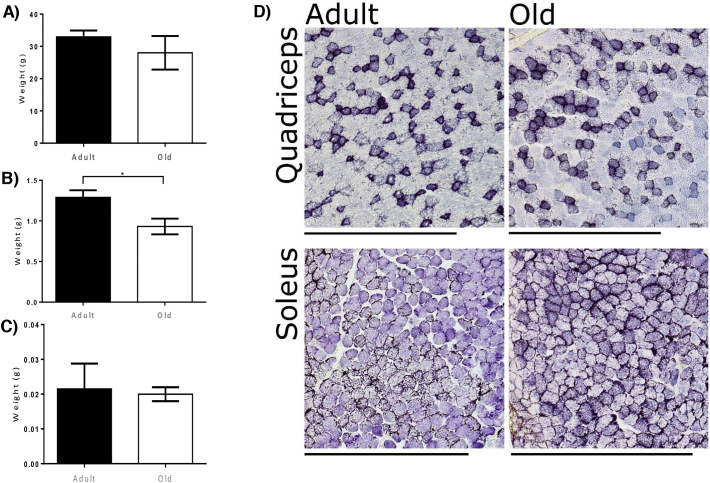
Body A) and Tissue Weights of B) Quadriceps and C) Soleus, D) Representative SDH staining of sections from adult and old quadriceps and soleus muscles (Scale bar represents 500 μm).

**Supplementary Fig. 1:**
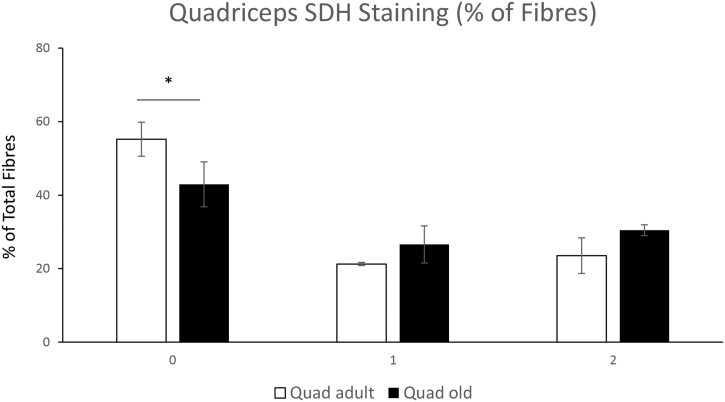
The % of SDH stained fibres from adult and old quadriceps tissue was calculated using a modified 3-point scale: 0 - unstained fibres; 1 – lightly stained fibres, 2 – dark stained fibres for semi quantification. The data shows % unstained/stained fibres of total fibres. All visible fibres in the section of the muscle were calculated. *N* = 3 biological replicates.

**Supplementary Fig. 2:**
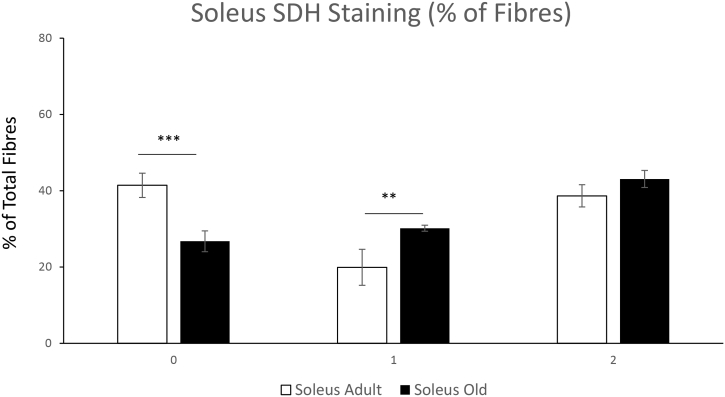
The % of SDH stained fibres from adult and old quadriceps tissue was calculated using a modified 3-point scale: 0 - unstained fibres; 1 – lightly stained fibres, 2 – dark stained fibres for semi quantification. The data shows % unstained/stained fibres of total fibres. All visible fibres in the section of the muscle were calculated. N = 3 biological replicates.

**Supplementary Fig. 3:**
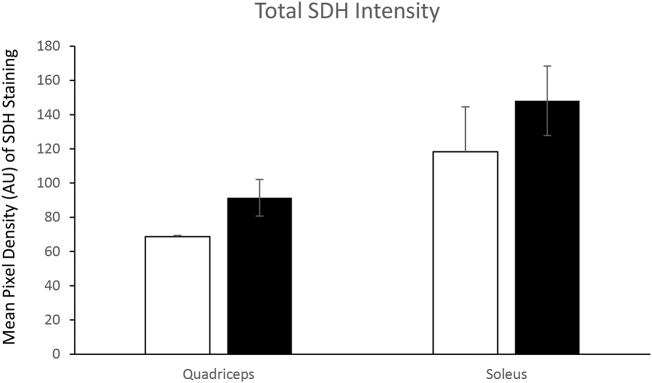
The total intensity of SDH staining of adult and old quadriceps and soleus tissue as measured by mean pixel intensity.

There was no significant decrease in overall body weight between adult and old mice. There was a significant reduction in quadriceps mass in old mice but no change in soleus mass Fig. 1A, B & C. In order to determine whether there was any major shifts in the proportion of oxidative fibres with age we performed SDH staining on transverse sections of the quadriceps and soleus muscles from adult and old mice. The % of stained fibres of quadriceps and soleus are presented in Supplementary Fig. 1, Supplementary Fig. 2, total SDH staining intensity is presented in Supplementary Fig. 3. These images confirm the greater proportion of oxidative fibres in soleus compared with quadriceps muscles and also that aging increased the proportion of oxidative fibres in muscle from old mice compared with those from adult mice.

### 3.2 Label free proteomics reveal an increase in cytoskeletal proteins in quadriceps and decrease in mitochondrial proteins in soleus with age

**Fig. 2:**
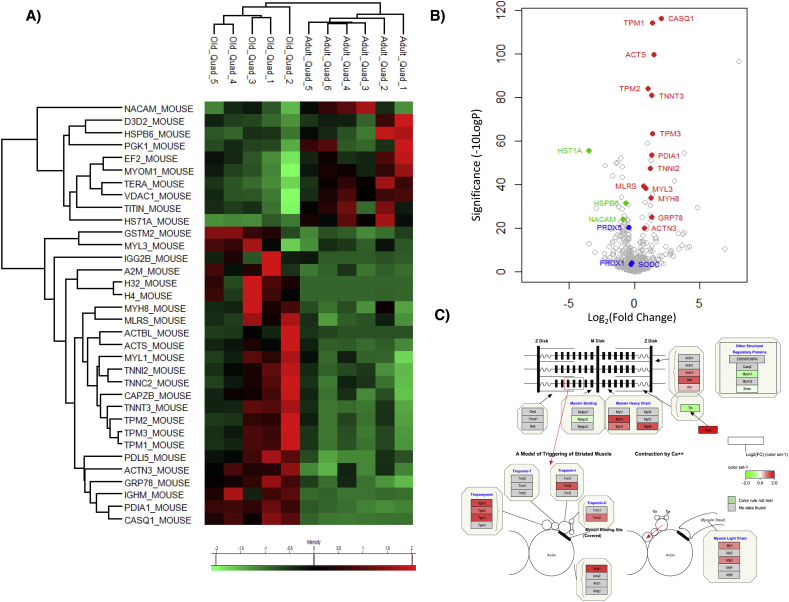
Label free proteomics of quadriceps from adult and old mice (A) Heatmap of significantly changed proteins (fold change >1.5 and –10logP > 20 equivalent to *P* < 0.01) (B) Volcano plot of quantified protein changes with age and significance, proteins highlighted in red are significantly increased with age, proteins highlighted in green are decreased with age (C) Pathway analysis of quantitative proteomic data reveals an upregulation of proteins involved in muscle contraction.

**Fig. 3:**
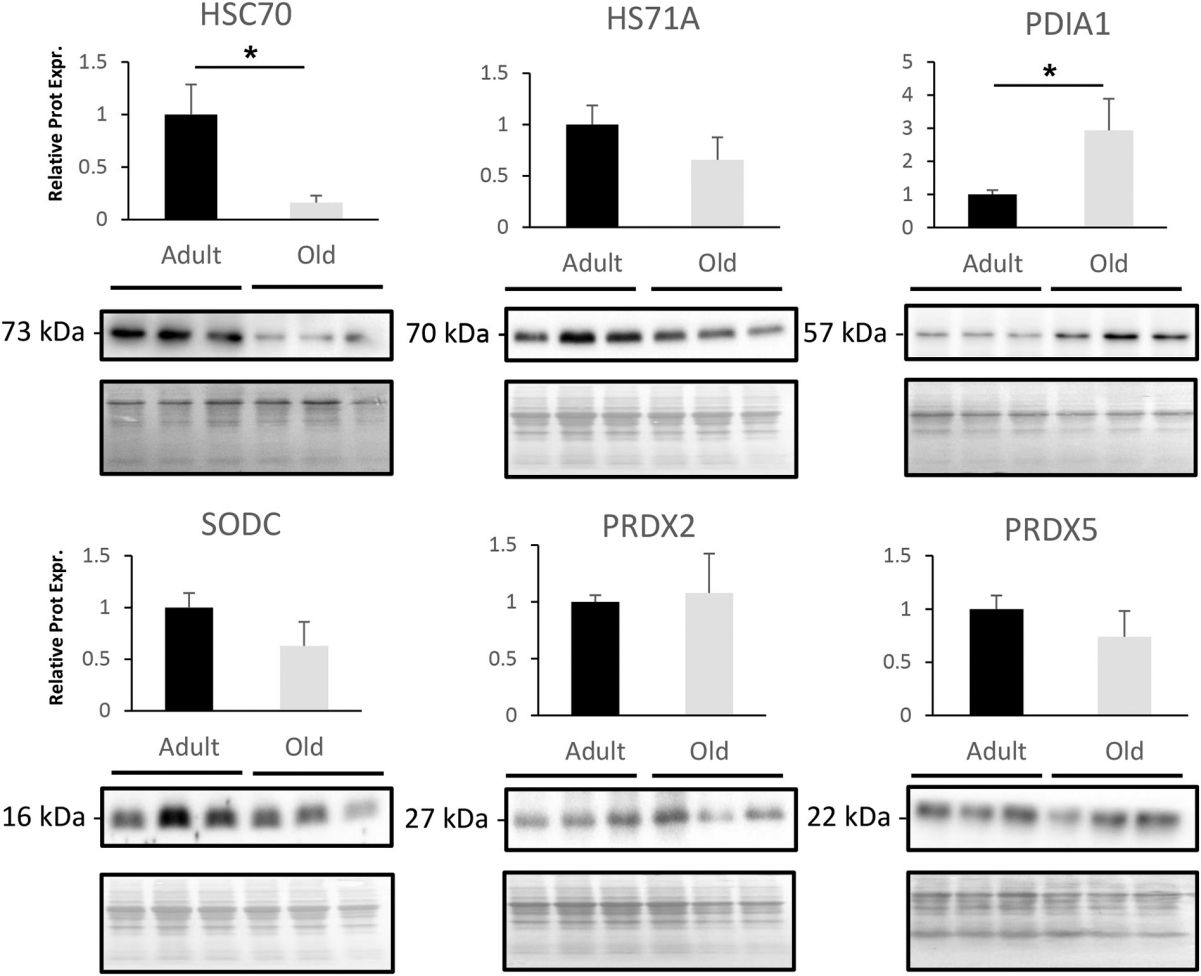
Western blot analysis and relative quantification of selected proteins from protein extracts of quadriceps protein extracts from adult and old mice (*N* = 3). *P < 0.05 using a two tailed unpaired student *t*-test. Corresponding Ponceau S stain of total protein on membranes were used as loading controls.

Label free proteomics was performed using Peaks7 quantification software and overall 946 proteins were quantified between the tissues from adult and old mice. Of the total number of identified proteins 434 were common to both quadriceps and soleus tissues, 205 were identified only in quadriceps and 307 only in soleus. Proteins were considered as significantly changed between adult and old mice in each tissue using the parameters *P* < 0.01 (−10 Log *P* ≥ 20), fold change ≥1.5 and with at least 3 unique peptides. The label free quantitative proteomic data from this study has been deposited in Mendeley data repository with doi:10.17632/x9wxcnc67r.1. In Quadriceps, 639 proteins were identified (Supplementary File 1 in data repository) of which 34 were considered significantly changed between adult and old mice, 23 up regulated and 11 down regulated with age (Fig. 2A and B). In quadriceps from old mice there was an increased abundance of cytoskeletal proteins including Tropomyosin (TPM1 and TPM2), Troponin (TNNI2 and TNNT3), Myosin (MYL3 and MYH8) and Actin (ACTS). There was a decrease in chaperone proteins including HS71A and HSPB6. Interestingly NACAM (Nascent polypeptide-associated complex subunit alpha, muscle-specific form or skNAC) was decreased and this protein is involved in the expression of genes in development of myotubes and assembly of thick and thin myofibril assembly [32]. Pathway analysis of the quantitative proteomic data confirmed an increase in proteins involved in the muscle contractile apparatus and cytoskeletal proteins (Fig. 2C). We selected a number of proteins that showed a significant differential expression for analysis by immunoblotting including Heat shock cognate 71 kDa (HSC70) and Protein disulfide isomerase (PDI) that indicated similar changes to the label free proteomic data, but no significant change in Heat shock protein 70 kDa (HS71A) was detected (Fig. 3). No significant changes were detected in antioxidant proteins with age using label free proteomics and immunoblotting confirmed these results for PRDX2, PRDX5 and SODC (Fig. 3) and highlighted in blue in the volanco plot of all label free proteomic data (Fig. 2B).

**Fig. 4:**
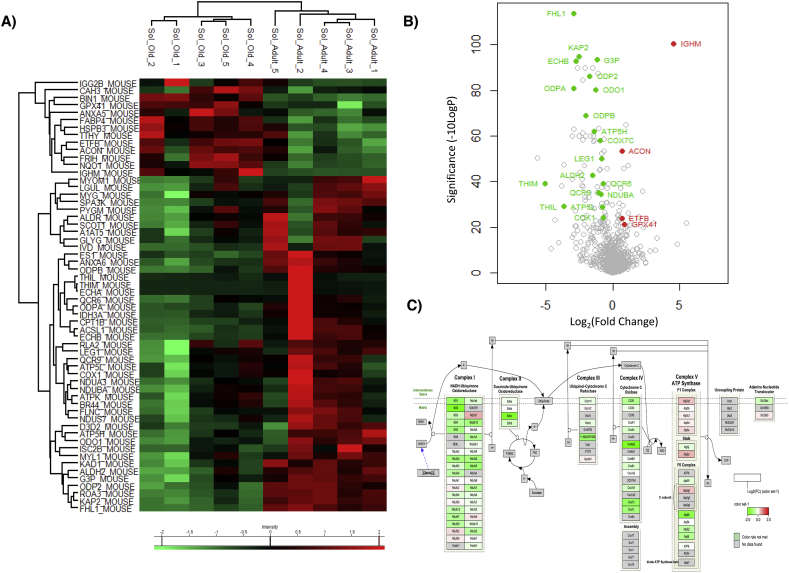
Label free proteomics soleus from adult and old mice, (A) Heatmap of significantly changed proteins with fold change >1.5 and –10logP > 20 equivalent to *P* < 0.01 (B) Volcano plot of quantified protein changes with age and significance, proteins highlighted in red are significantly increased with age and proteins highlighted in green are decreased with age. Proteins highlighted in blue were antioxidant proteins that did not significantly change in abundance and were selected for immunoblotting. (C) Pathway analysis of quantitative proteomic data using PathVisio reveals a downregulation of proteins involved in mitochondrial electron transport chain.

In soleus muscle from adult and old mice 741 proteins were quantified (Supplementary File 2 in data repository). There was a significant change in abundance of 58 proteins between adult and old mice, 45 proteins had decreased content and 13 proteins increased content in muscle from old mice (Fig. 4A and B). Proteins that decreased with age included a large number of mitochondrial proteins and proteins involved in the electron transport chain Fig. 4C. A reduced respiratory capacity with age has previously been demonstrated in soleus muscle [33]. Comparing the significant protein changes between both sets of tissues, 2 proteins had decreased protein content in both muscle types with age (3 2-trans-enoyl-CoA isomerase mitochondrial, Dci and Myomesin-1, Myom1) and 2 proteins had increased protein content (Ig mu chain C, Igh 6 and Ig gamma-2B chain C region, Igh 3). One protein, Myosin light chain 1/3 skeletal muscle isoform, (Myl1) had increased protein content in quadriceps and decreased protein content in soleus with age.

### 3.3 Age-related changes in redox state of Cys residues in metabolic and cytoskeletal proteins in both quadriceps and soleus

**Supplementary Fig. 4:**
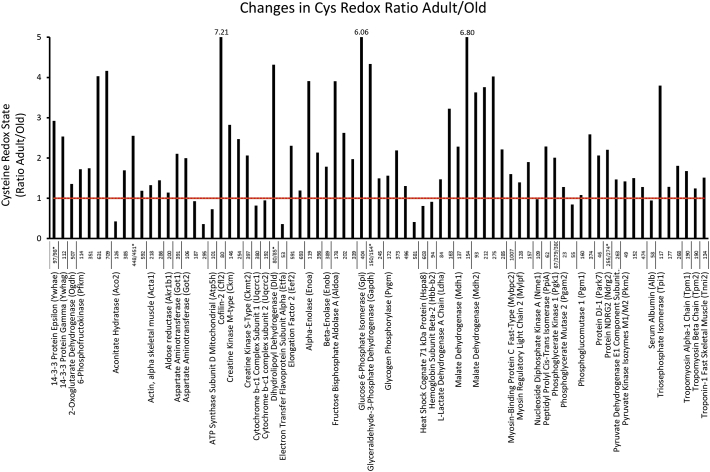
Changes in red/reversibly oxidised ratio of individual Cys residues with age in quadriceps muscle, ratio change <1 reflects increased reversible oxidation with age, ratio > 1 reflects decreased cysteine oxidation with age.

**Supplementary Fig. 5:**
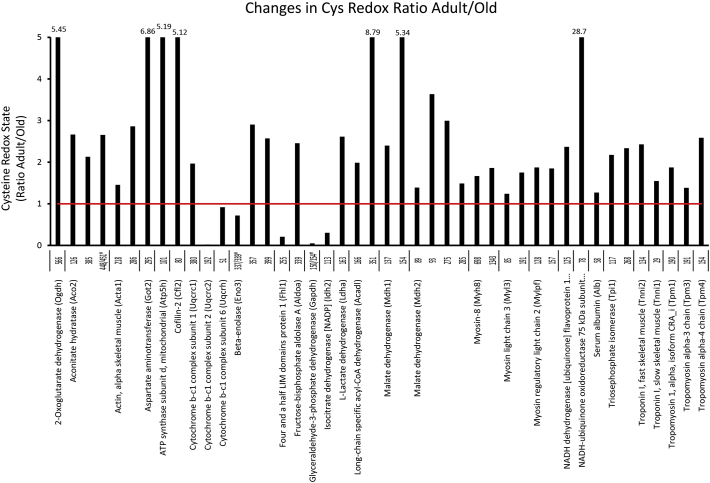
Changes in red/reversibly oxidised ratio of individual Cys residues with age in soleus skeletal muscle, ratio change <1 reflects increased reversible oxidation with age, ratio > 1 reflects decreased cysteine oxidation.

Cys containing peptides that were identified with high confidence (Mascot score > 20) labelled with both *d*0 and *d*5 NEM were selected for targeted analysis using the Skyline software. In the Quadriceps 75 redox Cys peptides from 45 proteins were quantified using Skyline. The list of the proteins and changes in the redox state of specific Cys residues with age are shown in Table 1, label free quantification at the protein level is included and proteins that significantly changed in abundance are in bold. In Soleus 44 redox peptides from 30 proteins were quantified using Skyline, Table 2. The changes with age in the ratio of red/rev oxidised redox state of the Cys residues for quadriceps and soleus tissues are presented in Supplementary Fig. 4, Supplementary Fig. 5.

The redox state of peptides has been found to both decrease and increase with age, indicating a more oxidised or reduced state of the Cys residue respectively. The redox state of 25 peptides from 17 proteins were quantified from both quadriceps and soleus, 5 of these Cys residues had an increased oxidation state with age in quadriceps and more reduced redox state in soleus, Aconitase Cys126, mitochondrial Aspartate Aminotransferase Cys295, mitochondrial ATP synthase subunit d Cys101, Cytochrome b-c1 complex subunit 1 Cys 380 and Serum albumin Cys58. The peptide containing the catalytic site of glyceraldehyde dehydrogenase Cys150/154 was more oxidised with age in the soleus and more reduced in quadriceps. There were no significant changes in abundance for these proteins with age and the muscle specific changes in redox state of individual Cys residues could indicate an altered intracellular redox environment. The changes in the redox state of individual Cys residues and the potential effects on protein activity need to be considered on an individual basis. For instance Cys residues involved in the co-ordination of iron in Fe-S proteins have been identified such as aconitase (Cys126, 286, 385, 448/451) and isocitrate dehydrogenase (Cys113). Metabolic enzymes involved in glycolysis and electron transport chain had substantial shifts in the redox state of Cys residues such as pyruvate kinase (Cys49, 152, 474), enolase alpha (Cys119, 399) and beta (Cys389), aldolase (Cys178, 202), malate dehydrogenase 1 (Cys137, 154) and 2 (Cys93, 212, 275, 285), ATP synthase (Cys101) and cytochrome *b*-c1 complexes 1 (Cys380), 2 (Cys192) and 6 (Cys80/85). There were also changes in the redox state of many of the cytoskeletal proteins that form part of contractile machinery for excitation contraction coupling and force generation; actin (Cys218, 286), myosin light chain 3 (Cys85, 191), myosin-8 (Cys698, 1343), myosin binding protein C (Cys1007), myosin regulatory light chain 2 (Cys128, 157), tropomyosin (Cys190), troponin I fast (Cys134) and slow (Cys29). Many of these sarcomeric proteins have previously been identified as modified by S-nitrosylation including myosin, actin, myosin-binding protein C, troponin C and I [34]. Cys134 from Troponin I in both quadriceps and soleus had a shift towards a more reduced state with age, this Cys residue has been reported to be both S-nitrosylated and S-glutathionylated in a recent study with opposite effects on protein function, S-nitrosylation decreases and S-glutathionylation of Cys134 increases the calcium-sensitivity of contractile machinery [35]. The technique used here does not allow the discrimination of specific reversible redox modifications but it does highlight the sensitivity of the approach for the detection of key Cys residues that affect protein activity and contractility.

**Table 1:** Proteins detected in quadriceps tissue containing redox sensitive Cys residues including the relative quantification of oxidative state of susceptible Cys residues and changes in the redox ratio with age. Relative protein abundance is included and proteins in bold change significantly with age.

Accession	Protein	Adult:Old	Significance (–10logP)	Peptide	Cysteine	Adult Red/Ox	Old Red/Ox	Change with age
P62259	14-3-3 Protein epsilon (Ywhae)	1.00:1.16	6.06	LI**CC**DILDVLDK	97/98*	7.69	22.47	2.92
P61982	14-3-3 Protein gamma (Ywhag)	1.00:1.04	6.62	N**C**SETQYESK	112	14.21	36.02	2.53
Q60597	2-Oxoglutarate dehydrogenase (Ogdh)	1.00:1.12	2.15	DVVVDLV**C**YR	507	0.53	0.71	1.35
P47857	6-Phosphofructokinase (Pfkm)	1.00:0.70	8.36	GITNL**C**VIGGDGSLTGADTFR	114	5.63	9.68	1.72
				LPLME**C**VQVTK	351	8.65	15.08	1.74
				**C**NENYTTDFIFNLYSEEGK	631	5.22	21.04	4.03
				IFANTPDSG**C**VLGMR	709	3.37	14.03	4.16
Q99KI0	Aconitate hydratase (Aco2)	1.00:1.00	1.85	VAVPSTIH**C**DHLIEAQVGGEK	126	61.41	26.17	0.43
				VGLIGS**C**TNSSYEDMGR	385	0.66	1.12	1.70
				DVGGIVLANA**C**GP**C**IGQWDR	448/451*	0.13	0.33	2.55
				**C**TTDHISAAGPWLK	592	19.33	22.92	1.19
**O88990**	**Actin, alpha skeletal muscle (Acta1)**	**1.00:2.82**	**99.81**	L**C**YVALDFENEMATAASSSSLEK	218	4.65	6.16	1.33
				**C**DIDIR	286	4.36	6.32	1.45
P45376	Aldose reductase (Akr1b1)	1.00:0.74	15.33	LIEY**C**HSK	200	0.71	0.81	1.14
P05201	Aspartate aminotransferase (Got1)	1.00:1.10	4.28	INM**C**GLTTK	391	3.54	7.45	2.10
P05202	Aspartate aminotransferase (Got2)	1.00:1.13	4.92	EYLPIGGLAEF**C**K	106	5.36	10.69	1.99
				T**C**GFDFSGALEDISK	187	4.31	3.99	0.93
				VGAFTVV**C**K	295	0.16	0.06	0.36
Q9DCX2	ATP synthase dubunit d mitochondrial (Atp5h)	1.00:1.27	8.20	S**C**AEFVSGSQLR	101	51.73	37.60	0.73
P45591	Cofilin-2 (Cfl2)	1.00:1.07	5.60	LLPLND**C**R	80	1.90	13.71	7.21
P07310	Creatine kinase M-type (Ckm)	1.00:1.14	3.01	GYTLPPH**C**SR	146	6.40	18.06	2.82
				F**C**VGLQK	254	11.50	28.41	2.47
Q6P8J7	Creatine kinase S-type (Ckmt2)	1.00:0.95	5.14	SEVELVQIVIDGVNYLVD**C**EK	397	3.60	7.42	2.06
Q9CZ13	Cytochrome b-c1 complex subunit 1 (Uqcrc1)	1.00:0.92	9.70	L**C**TSATESEVTR	380	1.96	1.60	0.82
Q9DB77	Cytochrome b-c1 complex subunit 2 (Uqcrc2)	1.00:1.09	6.20	NALANPLY**C**PDYR	192	0.02	0.02	0.95
O08749	Dihydrolipoyl dehydrogenase (Dld)	1.00:1.99	8.53	NETLGGT**C**LNVG**C**IPSK	80/85*	71.21	307.42	4.32
Q99LC5	Electron transfer flavoprotein subunit alpha (Etfa)	1.00:0.85	12.77	LGGEVS**C**LVAGTK	53	15.40	5.53	0.36
**P58252**	**Elongation factor 2 (Eef2)**	**1.00:0.59**	**25.73**	ETVSEESNVL**C**LSK	591	2.86	6.59	2.30
				EGAL**C**EENMR	693	14.47	17.27	1.19
P17182	Alpha-enolase (Enoa)	1.00:1.32	1.46	FGANAILGVSLAV**C**K	119	2.14	8.36	3.91
				TGAP**C**R	398	4.18	8.93	2.14
P21550	Beta-enolase (Enob)	1.00:0.89	8.60	SGETEDTFIADLVVGL**C**TGQIK	389	3.60	6.42	1.79
P05064	Fructose bisphosphate aldolase A (Aldoa)	1.00:1.04	3.97	YASI**C**QQNGIVPIVEPEILPDGDHDLK	178	0.53	2.08	3.91
				**C**QYVTEK	202	2.50	6.56	2.62
				ALANSLA**C**QGK	339	5.77	11.37	1.97
P06745	Glucose 6-phosphate isomerase (Gpi)	1.00:1.25	6.57	MIP**C**DFLIPVQTQHPIR	404	0.48	2.89	6.06
P16858	Glyceraldehyde-3-phosphate dehydrogenase (Gapdh)	1.00:1.02	8.18	IVSNAS**C**TTN**C**LAPLAK	150/154*	127.86	554.21	4.33
				VPTPNVSVVDLT**C**R	245	17.90	26.74	1.49
Q9WUB3	Glycogen phosphorylase (Pygm)	1.00:0.98	10.01	I**C**GGWQMEEADDWLR	172	6.47	10.09	1.56
				T**C**AYTNHTVLPEALER	373	4.81	10.53	2.19
				WLVL**C**NPGLAEVIAER	496	14.05	18.31	1.30
				QLLN**C**LHIITLYNR	581	30.97	12.73	0.41
P63017	Heat shock cognate 71 kDa protein (Hspa8)	1.00:0.79	11.10	V**C**NPIITK	603	10.88	8.79	0.81
P02089	Hemoglobin subunit beta-2 (Hbb-b2)	1.00:1.54	5.99	GTFASLSELH**C**DK	94	100.34	91.44	0.91
**P06151**	**l-Lactate dehydrogenase A chain (Ldha)**	**1.00:0.71**	**23.73**	DY**C**VTANSK	84	13.62	20.01	1.47
				VIGSG**C**NLDSAR	163	1.33	4.29	3.23
P14152	Malate dehydrogenase (Mdh1)	1.00:1.06	6.17	VIVVGNPANTN**C**LTASK	137	15.57	35.51	2.28
				ENFS**C**LTR	154	0.94	6.36	6.80
P08249	Malate dehydrogenase (Mdh2)	1.00:0.76	10.78	G**C**DVVVIPAGVPR	93	0.69	2.52	3.63
				TIIPLISQ**C**TPK	212	22.06	82.92	3.76
				EGVVE**C**SFVQSK	275	2.44	9.84	4.02
				ETE**C**TYFSTPLLLGK	285	4.35	9.62	2.21
Q5XKE0	Myosin-binding protein C fast-type (Mybpc2)	1.00:0.81	12.24	IFSENI**C**GLSDSPGVSK	1007	48.64	77.79	1.60
**P97457**	**Myosin regulatory light chain 2 (Mylpf)**	**1.00:1.63**	**39.47**	QFLEELLTTQ**C**DR	128	16.04	22.38	1.40
				NI**C**YVITHGDAK	157	7.32	13.91	1.90
P15532	Nucleoside diphosphate kinase A (Nme1)	1.00:0.59	12.77	GDF**C**IQVGR	109	14.89	14.54	0.98
P17742	Peptidyl prolyl cis-trans isomerase (PpiA)	1.00:0.90	2.84	IIPGFM**C**QGGDFTR	62	2.52	5.76	2.29
**P09411**	**Phosphoglycerate kinase 1 (Pgk1)**	**1.00:0.59**	**23.68**	G**C**ITIIGGGDTAT**CC**AK	367/379/380*	47.50	95.40	2.01
**O70250**	**Phosphoglycerate mutase 2 (Pgam2)**	**1.00:0.71**	**30.92**	FCGWFDAELSEK	23	7.19	9.20	1.28
				IEFDICYTSVLK	55	1.31	1.11	0.85
Q9D0F9	Phosphoglucomutase 1 (Pgm1)	1.00:0.75	14.98	TIEEYAI**C**PDLK	160	15.52	16.76	1.08
				LSL**C**GEESFGTGSDHIR	374	3.25	8.42	2.59
Q99LX0	Protein DJ-1 (park7)	1.00:0.81	6.52	DPVQ**C**SR	46	8.50	17.53	2.06
Q9QYG0	Protein NDRG2 (Ndrg2)	1.00:0.83	9.86	**C**PVMLVVGDQAPHEDAVVE**C**NSK	255/274*	14.57	32.14	2.21
Q9D051	Pyruvate dehydrogenase E1 component subunit beta (Pdhb)	1.00:0.82	6.78	EGIE**C**EVINLR	263	6.80	9.97	1.47
P52480	Pyruvate kinase isozymes M1/M2 (Pkm2)	1.00:0.98	3.86	NTGII**C**TIGPASR	49	12.06	17.11	1.42
				**C**DENILWLDYK	152	5.75	8.62	1.50
				GIFPVL**C**K	474	23.16	29.64	1.28
P07724	Serum albumin (Alb)	1.00:1.25	9.00	**C**SYDEHAK	58	2.95	2.78	0.94
P17751	Triosephosphate isomerase (Tpi1)	1.00:0.84	8.68	IAVAAQN**C**YK	117	0.63	2.40	3.80
				VSHALAEGLGVIA**C**IGEK	177	9.15	11.75	1.28
				IIYGGSVTGAT**C**K	268	7.29	13.17	1.81
**P58771**	**Tropomyosin alpha-1 chain (Tpm1)**	**1.00:2.60**	**115.68**	**C**AELEEELK	190	5.65	9.46	1.68
**P58774**	**Tropomyosin beta chain (Tpm2)**	**1.00:2.04**	**84.11**	**C**GDLEEELK	190	6.34	7.89	1.25
**P13412**	**Troponin-1 fast skeletal muscle (Tnni2)**	**1.00:2.32**	**52.67**	V**C**MDLR	134	6.79	10.27	1.51

* Signifies that 2 or more Cys residues present in tryptic peptide.

**Table 2:** Proteins detected in soleus tissue containing redox sensitive Cys residues including the relative quantification of oxidative state of susceptible Cys residues and changes in the redox ratio with age. Relative protein abundance is included and proteins in bold changed significantly with age.

Accession	Protein	Adult:Old	Significance (–10logP)	Peptide	Cysteine	Adult Red/Ox	Old Red/Ox	Change with age
**Q60597**	**2-Oxoglutarate dehydrogenase (Ogdh)**	**1.00:0.41**	**80.24**	I**C**EEAFTR	566	4.02	21.91	5.45
**Q99KI0**	**Aconitate hydratase (Aco2)**	**1.00:1.60**	**53.50**	VAVPSTIH**C**DHLIEAQVGGEK	126	4.35	11.59	2.66
				VGLIGS**C**TNSSYEDMGR	385	1.85	3.95	2.13
				DVGGIVLANA**C**GP**C**IGQWDR	448/451*	3.18	8.43	2.66
O88990	Actin, alpha skeletal muscle (Acta1)	1.00:1.17	12.03	L**C**YVALDFENEMATAASSSSLEK	218	11.14	16.23	1.46
				**C**DIDIR	286	13.81	39.52	2.86
P05202	Aspartate aminotransferase (Got2)	1.00:1.02	7.39	VGAFTVV**C**K	295	1.90	13.05	6.86
**Q9DCX2**	**ATP synthase subunit d, mitochondrial (Atp5h)**	**1.00:0.38**	**61.87**	S**C**AEFVSGSQLR	101	24.99	129.81	5.19
P45591	Cofilin-2 (Cfl2)	1.00:1.43	18.30	LLPLND**C**R	80	14.54	74.39	5.12
Q9CZ13	Cytochrome b-c1 complex subunit 1 (Uqcrc1)	1.00:0.89	8.96	L**C**TSATESEVTR	380	23.64	46.49	1.97
Q9DB77	Cytochrome b-c1 complex subunit 2 (Uqcrc2)	1.00:1.04	3.16	NALANPLY**C**PDYR	192	4833.44	90.84	0.02
**P99028**	**Cytochrome b-c1 complex subunit 6 (Uqcrh)**	**1.00:0.61**	**39.21**	ERLEL**C**DNR	51	25.97	23.86	0.92
P21550	Beta-enolase (Eno3)	1.00:0.79	14.85	A**C**N**C**LLLK	337/339*	84.66	60.82	0.72
				VNQIGSVTESLQA**C**K	357	7.14	20.73	2.91
				TGAP**C**R	399	8.89	22.85	2.57
**P97447**	**Four and a half LIM domains protein 1 (Fhl1)**	**1.00:0.13**	**113.70**	**C**SVNLANKR	255	476.66	99.38	0.21
**P05064**	**Fructose-bisphosphate aldolase A (Aldoa)**	**1.00:1.43**	**23.11**	ALANSLA**C**QGK	339	21.69	53.27	2.46
**P16858**	**Glyceraldehyde-3-phosphate dehydrogenase (Gapdh)**	**1.00:0.44**	**93.31**	IVSNAS**C**TTN**C**LAPLAK	150/154*	5524.28	278.52	0.05
P54071	Isocitrate dehydrogenase [NADP] (Idh2)	1.00:0.93	7.60	**C**ATITPDEAR	113	0.02	0.01	0.30
P06151	l-Lactate dehydrogenase (Ldha)	1.00:0.68	25.24	VIGSG**C**NLDSAR	163	22.79	59.57	2.61
P51174	Long-chain specific acyl-CoA dehydrogenase (Acadl)	1.00:1.08	10.15	**C**IGAIAMTEPGAGSDLQGVR	166	7.54	14.97	1.99
				AFVDS**C**LQLHETK	351	2.73	23.99	8.79
P14152	Malate dehydrogenase (Mdh1)	1.00:0.97	5.01	VIVVGNPANTN**C**LTASK	137	9.02	21.65	2.40
				ENFS**C**LTR	154	8.23	43.95	5.34
P08249	Malate dehydrogenase (Mdh2)	1.00:1.01	3.84	GYLGPEQLPD**C**LK	89	27.10	37.69	1.39
				G**C**DVVVIPAGVPR	93	4.48	16.30	3.63
				EGVVE**C**SFVQSK	275	13.51	40.48	3.00
				ETE**C**TYFSTPLLLGK	285	24.59	36.59	1.49
P13542	Myosin-8 (Myh8)	1.00:1.22	3.97	**C**NGVLEGIR	698	43.95	73.27	1.67
				HD**C**DLLR	1343	24.04	44.75	1.86
**P09542**	**Myosin light chain 3 (Myl3)**	**1.00:1.17**	**20.73**	ITYGQ**C**GDVLR	85	54.39	67.53	1.24
				LMAGQEDSNG**C**INYEAFVK	191	15.56	27.26	1.75
P97457	Myosin regulatory light chain 2 (Mylpf)	1.00:0.73	45.56	QFLEELLTTQ**C**DR	128	27.50	51.56	1.87
				NI**C**YVITHGDAK	157	23.14	42.82	1.85
Q91YT0	NADH dehydrogenase [ubiquinone] flavoprotein 1 (Ndufv1)	1.00:1.01	5.73	YLVVNADEGEPGT**C**K	125	295.18	699.84	2.37
Q91VD9	NADH-ubiquinone oxidoreductase 75 kDa subunit (Ndufs1)	1.00:0.87	9.42	M**C**LVEIEK	78	115.96	3328.04	28.70
P07724	Serum albumin (Alb)	1.00:0.90	12.32	**C**SYDEHAK	58	3.83	4.87	1.27
P17751	Triosephosphate isomerase (Tpi1)	1.00:0.98	14.07	IAVAAQN**C**YK	117	731.14	1591.58	2.18
				IIYGGSVTGAT**C**K	268	19.41	45.36	2.34
**P13412**	**Troponin I, fast skeletal muscle (Tnni2)**	**1.00:0.81**	**39.05**	V**C**MDLR	134	18.38	44.64	2.43
Q9WUZ5	Troponin I, slow skeletal muscle (Tnni1)	1.00:0.91	12.86	E**C**WEQEHEER	29	85.64	132.57	1.55
P58771	Tropomyosin 1, alpha, isoform CRA_i (Tpm1)	1.00:1.00	7.40	**C**AELEEELK	190	16.71	31.32	1.87
P21107	Tropomyosin alpha-3 chain (Tpm3)	1.00:1.19	13.81	**C**SELEEELK	191	55.27	76.49	1.38
Q6IRU2	Tropomyosin alpha-4 chain (Tpm4)	1.00:1.77	5.77	**C**GDLEEELK	154	14.01	36.23	2.59

* Signifies that 2 or more Cys residues present in tryptic peptide.

**Fig. 5:**
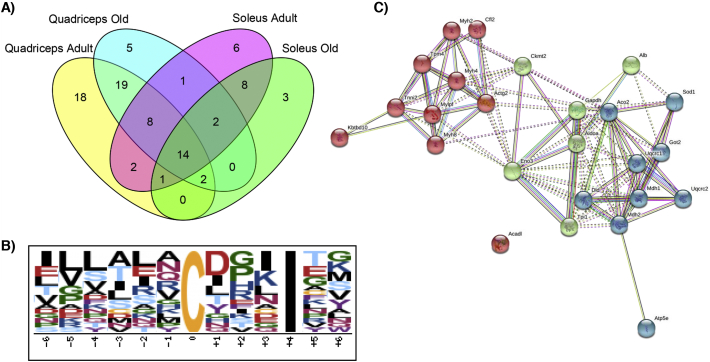
Redox changes between Quadriceps and Soleus a) numbers of redox proteins detected in the different groups, in quadriceps adult muscle there were 64 compared to 53 in old and in soleus there were 28 redox proteins in old compared to 42 in adult. B) To determine if there was a consensus motif surrounding the redox sensitive Cys residues identified in this analysis we used Motif-X. A protein blast against the Mus proteome was performed using the amino acid sequences −6 to +6 of the redox sensitive Cys residues identified to determine potential motifs in surrounding amino acids. The highest scoring motif (6.14 and *P* < 0.000001) included Isoleucine (I) at a position +4 from the identified redox sensitive peptides using Motif-X. C) To determine if there was an over representation of particular metabolic pathways or protein-protein interactions of identified proteins containing redox sensitive Cys residues we used String-DB. Identified redox sensitive Cys containg proteins revealed a highly interconnected network and an enrichment for proteins involved in the generation of precursor metabolites and energy (GO:0006091, FDR value 2.06e^−09^).

The number of proteins identified as containing redox sensitive Cys residues decreases with age in both quadriceps and soleus (Fig. 5A), which may indicate a reduced redox flexibility in both these tissues with age. It should be pointed out that not all of the redox sensitive peptides initially identified were quantifiable using Skyline and were not included in Table 1, Table 2. Using the identified redox sensitive Cys residues we searched for possible conserved redox motifs or over representation of certain amino acids in the mouse proteome. The highest scoring motif from a Motif-X blast [30] had an Isoleucine residue on the +4 C-terminal side of the redox sensitive Cys (Fig. 5B). A String-DB analysis [31] of identified redox proteins (Fig. 5C) indicate that peptides identified in both tissues are enriched for proteins involved in the generation of precursor metabolites and energy (GO:0006091, FDR 2.06^−09^).

The data presented here combined a label free proteomic approach with differential Cys labelling to analyse changes in the global proteome and for the first time, identified how the redox state of sensitive Cys residues change with age in two metabolically distinct skeletal muscles. Overall the global proteomic changes were found to be fibre type dependent with significant increases in cytoskeletal proteins in quadriceps and significant decreases in electron transport chain proteins in soleus but the redox proteome was more preserved between tissues with specific changes in redox state of metabolic and cytoskeletal proteins.

## 4 Discussion

The results of this study describe the effects of age on the protein composition of two distinct muscle groups. There was an increase in cytoskeletal proteins in glycolytic quadriceps muscle and a decrease in mitochondrial proteins involved in electron transport chain in more oxidative soleus muscle. Analysis of the redox state of sensitive Cys residues indicate that age-related changes are identified predominantly in cytoskeletal and metabolic proteins in both muscles. This study provides further information on the effects of age on skeletal muscle groups with distinct susceptibility to age-related atrophy. In humans the age-related loss of skeletal muscle can result in frailty and loss of independence, and is a major factor in contributing to the co-morbidities of many diseases. One of the most effective therapies to delay age-related atrophy of skeletal muscle mass and function is regular exercise while disuse can accelerate the loss of muscle mass [36,37]. Contracting skeletal muscle generates ROS [[11], [12], [13]] that are thought to have a vital role in the beneficial responses to exercise and aid in the repair of injured skeletal muscle [15,16,38,39]. Previous studies using rodent models have shown that the response to redox generated signals for the activation of key transcription factors such as NFκB and Hsf-1 are blunted in muscle from old mice and there is a chronic activation of the inflammatory response [20].

Skeletal muscle that contains higher proportions of glycolytic fibres appear to be more susceptible to age-related atrophy [[3], [4], [5]]. Our results indicate that quadriceps had a reduced muscle mass and increased SDH staining with age, no significant decrease in muscle mass was detected in soleus but there was an increased proportion of SDH stained fibres. Aging results in a reduced respiratory capacity of rat soleus (mainly type I fibres) and glycolytic (type IIB and IIA) from white and red portions of Vastus lateralis [4]. Fast to slow fibre type remodelling with age has been reported, in particular a shift from IIB to IIX fibres in rodent fast twitch muscles such as the tibialis anterior [5] and Type IIA to Type I in plantaris [4,40]. In soleus it has also been reported that there is an increase in single muscle fibres that have increased co-expression of myosin heavy chain isoforms in older rats as a result of the reinnervation of denervated fibres [41]. The increased SDH staining in muscle fibres as representative of Krebs cycle mitochondrial activity could be a compensatory response, therefore to investigate the intracellular changes in the metabolically distinct skeletal muscles a global and redox proteomic approach was undertaken.

Global proteomic approaches that have utilised 2D electrophoresis including differential in gel electrophoresis [[42], [43], [44]] and gel-free approaches [45,46] to analyse the effects of age on skeletal muscles from rodents have reported similar changes in glycolytic, mitochondrial and cytoskeletal proteins with age as described in this study. Alterations in the proteome have been confirmed in more detail in a proteomic study of slow and fast human skeletal muscle single fibres isolated from biopsies of young (22–27) and older (67–75) individuals [47]. In a previous study examining the effects of age on gastrocnemius muscle, an increase in the abundance of cytoskeletal proteins and a decrease in metabolic proteins was also reported [21]. However, in the gastrocnemius, the increase in cytoskeletal proteins in response to age was not as dramatic as what we detected in quadriceps nor the decrease in mitochondrial proteins comparable to what was observed in soleus, which may reflect the more heterogeneous fibre type content. One protein Ig mu chain C increased in abundance across all three muscles with age, while Myl1 increased in abundance in quadriceps and decreased in soleus, also had increased abundance in gastrocnemius muscle in response to age [21]. The greater susceptibility of fast fibres to age-related atrophy could be related to the higher glycolytic flux within fast fibres similar to the metabolic reprogramming in tumour cells [48] affecting ROS generation and downstream pathways including autophagy and the proteasome [49]. It has also been suggested that lower mitochondrial protein turnover in fast fibres could result in reduced mitochondrial quality and increased susceptibility to proteasomal degradation [33]. Higher mitochondrial turnover in slow fibres may offer greater protection against dysfunctional metabolic pathways related to sarcopenia. However, a decrease in the abundance of proteins involved in mitochondrial electron transport proteins with age in soleus yet an increase in mitochondrial SDH activity could be a compensatory mechanism for incomplete metabolism by oxidative phosphorylation.

In comparison to the global changes in proteome abundance, the changes in the redox proteome appeared to be preserved between muscles. In this study, 25 of the Cys containing peptides that had an altered redox state with age were common to both quadriceps and soleus (Table 1, Table 2). In comparison to a previous study of the effects of age on the gastrocnemius muscle 23 of these 25 Cys residues were also reported as having an altered redox state [21]. The transient redox state of key individual Cys residues has an important role in skeletal muscle redox signalling, allowing redox sensitive proteins to rapidly respond to local changes in ROS concentrations. Analysis of individual Cys residues indicate that in a number of instances there was greater oxidation in the redox state with age but this was not always the case and often there were large shifts to a more reduced state in individual Cys residues from both tissues. However, there were 6 Cys residues quantified in both quadriceps and soleus where the redox state shifted in the opposite direction with age. Apart from glyceraldehyde dehydrogenase which requires the catalytic Cys residues in the active site (Cys150/154) to be in a reduced form [50], it was not possible to determine how these redox changes would affect protein activity from the current literature or structural data. Many proteins require their key redox sensitive Cys residues to be in a disulfide bond for functional activity e.g. triose phosphate isomerase and pyruvate kinase while others require the Cys to be reduced [50]. Therefore, it is necessary to consider the age-related changes in the redox state of specific Cys residues on an individual basis for each protein taking into account the location and function of the Cys residue.

A recent top down proteomic approach of rat slow and fast skeletal muscles identified changes with age in the phosphorylation status of a number of the cytoskeletal proteins identified as having an altered abundance and redox state in this study including Tropomyosin alpha and beta, Myosin regulatory light chain and Troponin I [51]. Troponin I was also found to have a reduced level of S-glutathionylation with age but the Cys residue modified was not reported [51]. The S-gluthathionylation and S-nitrosylation of Cys134 of Troponin I has been reported to increase and decrease respectively the Ca^2+^ sensitivity of this protein [35], highlighting the complexity of Cys specific redox modifications. In this study we identified Cys134 of Troponin I as having an altered redox state with age but could not discriminate the specific redox modifications and hence effects on Ca^2+^ sensitivity for contractile function, but our approach is sensitive for the identification of regulatory Cys residues. Similarly, oxidation of myosin residues will affect actin binding [52], identification and the relative quantification of the specific residues susceptible to oxidation may provide an indication of the events that lead to defects in mechanical function of skeletal muscle. Cross talk between Cys redox specific and PTMs such as phosphorylation in the context of protein abundance and subsequent effects on contractile function requires further study. It is unlikely that a protein is exclusively reduced or reversibly oxidised but in the approach used here we attempt to estimate the redox state of individual Cys residues. However, the sensitivity of this approach depends on the proportion and concentration of the peptide containing the Cys residue labelled, if it was identified with a high confidence in the initial search of raw data and with a consistent retention time and fragmentation across samples to allow targeted quantification using Skyline.

Overall, there was a reduction in the number of peptides that contain redox Cys residues labelled with both light and heavy isoform of NEM with age in quadriceps and soleus (Fig. 5A). In order to determine if there was particular amino acids over represented or potentially a conserved motif surrounding the redox sensitive Cys residues identified we used Motif-X [30]. Using the amino acid sequences −6 to +6 of the redox sensitive Cys, there was an over representation of Isoleucine at +4 C-terminal side of the redox sensitive Cys residues (Fig. 5B). Isoleucine does not contain reactive side chains but has an important role in protein stability and ligand binding. A String-DB analysis of identified proteins containing redox sensitive Cys residues indicates multiple interactions between proteins identified and an enrichment for proteins involved in precursor metabolites and energy generation (Fig. 5C), consistent with a previous study analyzing the effects of age on the gastrocnemius [21].

Previous studies have detected an increase in irreversible protein oxidation with age in skeletal muscle [53,54], the increase in proportion of specific Cys residues to a more reduced state seems to be counter intuitive in this intracellular environment. Many of the proteins identified as irreversibly modified appear to be highly abundant cytoskeletal proteins and metabolic enzymes that also have an increase in abundance with age. These proteins that could potentially act as a redox buffer inhibiting ROS induced redox signalling. The identification of highly abundant cytoskeletal and sarcomeric proteins also highlights one of the limitations of the data dependent acquisition mass spectrometry approach, as low abundant proteins and their parent ions may not get fragmented [55]. Sarcomeric and cytoskeletal proteins dominate the skeletal muscle proteome and can account for >50% of the proteome [56], thus comparing low to high abundant proteins can span up to seven orders of magnitude [47]. Increasing the sensitivity of detection by sample fractionation or data independent approaches would increase the accuracy of both detection and quantification of redox specific changes and for global label free proteomics.

## 5 Conclusions

The redox proteome was relatively preserved between metabolically distinct muscles. This is likely due to the nature of the regulatory nature of these residues in proteins involved in processes such as glycolysis, oxidative phosphorylation, translation, transcription and degradation [57]. It has previously been demonstrated that Cys residues have the most extreme conservation patterns across all of the amino acids, they are highly conserved when they play a fundamental role in protein activity in active sites or co-factor binding but poorly conserved otherwise [58]. Cysteine residues in myofilament proteins are generally conserved across different protein species and accessibility can depend on the contractile status of the muscle [59]. Nevertheless our observation of a reduction in available Cys residues for redox signalling during aging is likely to result in a reduced flexibility of the redox response and impair the ability of skeletal muscle to optimally respond and adapt to exercise.

## 6 Data repository

Quantitative proteomic data from this study has been deposited in Mendeley data repository with doi: 10.17632/x9wxcnc67r.1. Files include all protein detected, no of identified and unique peptides for individual proteins, intensities from each biological replicate, fold change including significance (–10logP).

The following are the supplementary data related to this article.

## Transparency document

Transparency document. Image 1
